# A multi-country outbreak of *Salmonella* Mbandaka linked to pre-cooked, frozen chicken meat in ready-to-eat products, Finland, 2022 to 2023

**DOI:** 10.2807/1560-7917.ES.2025.30.17.2400602

**Published:** 2025-05-01

**Authors:** Ana Cristina Gonzalez-Perez, Heidi Landgren, Anni Vainio, Wioleta Kitowska, Annika Pihlajasaari, Elina Leinonen, Henry Kuronen, Taru Lienemann, Heli Tapanainen, Niina E Kaartinen, Jelena Rjabinina, Jelena Sõgel, Ruska Rimhanen-Finne

**Affiliations:** 1 Finnish Institute for Health and Welfare, Helsinki, Finland; 2 ECDC Fellowship Programme, Public Health Microbiology path (EUPHEM), European Centre for Disease Prevention and Control (ECDC), Stockholm, Sweden; 3 ECDC Fellowship Programme, Field Epidemiology path (EPIET), European Centre for Disease Prevention and Control (ECDC), Stockholm, Sweden; 4 Finnish Food Authority, Helsinki and Kuopio, Finland; 5 Health Board, Tallinn, Estonia; 6 Agriculture and Food Board, Saku, Estonia

**Keywords:** *Salmonella* Mbandaka, multi-country, outbreak, food-borne, chicken, ready-to-eat products, case-case study, whole-genome sequencing, gastrointestinal

## Abstract

In May 2022, the Finnish Institute for Health and Welfare observed an increase in *Salmonella* Mbandaka cases. Whole genome sequencing (WGS) linked the outbreak strain to a previously reported strain in the United Kingdom. From April 2022 to January 2023, 97 cases were reported across 18 of Finland’s 21 hospital districts, with a median age of 27 years (range <1–74 years) and 61% female cases. Based on trawling interviews and national food consumption data, cases were more likely to have eaten at a restaurant or takeaway (odds ratio (OR) = 115; 95% confidence interval (CI): 52–256; p < 0.001) or consumed poultry products (OR = 28; 95% CI: 7–115; p < 0.001). A case–case study linked cases with consumption of ready-to-eat products containing chicken from a specific company (OR = 28; 95% CI: 1.9–1,361; p = 0.004). Traceback investigations identified a pre-cooked, frozen chicken meat product from a country outside the European Union as the likely source. *Salmonella* Mbandaka was isolated from cases and linked by WGS to this chicken meat product. Our findings highlight the potential health risk of pre-cooked chicken meat containing *Salmonella* and the value of case–case studies and product images to assist recall during food-borne outbreak investigations.

**Key public health message**

**What did you want to address in this study and why?**

*Salmonella* infections are a frequent cause of food-borne outbreaks in Europe. We investigated a widespread outbreak of *Salmonella* Mbandaka in Europe in order to identify the source and implement measures to prevent further cases and future outbreaks.

**What have we learnt from this study?**

The source of this outbreak, with over 200 cases, was traced to a pre-cooked, frozen chicken meat product from a country outside the European Union. Food business operators should visually inspect pre-cooked meat batches to ensure the meat is properly cooked before using it in ready-to-eat products.

**What are the implications of your findings for public health?**

Cross-border and cross-sectoral collaboration are critical for investigating multi-country outbreaks. By sharing epidemiological, traceback, and microbiological data, we identified the source of this widespread *Salmonella* Mbandaka outbreak.

## Background

Infection with non-typhoidal *Salmonella* (NTS) commonly manifests with sudden onset of diarrhoea, abdominal pain, fever, nausea, and vomiting (an overview of *Salmonella* infections is available from the ECDC (https://www.ecdc.europa.eu/en/salmonellosis) and the CDC (https://www.cdc.gov/salmonella/about/index.html)) [1]. More than 2,500 serotypes of *Salmonella* have been identified, of which a small proportion cause illness in humans [2]. *Salmonella* is the second most reported zoonotic agent in humans after *Campylobacter* and a major cause of food-borne outbreaks in the European Union and European Economic Area (EU/EEA) [3]. In Finland, the incidence of salmonellosis has decreased in the past 10 years and in 2021, it was the third most reported notifiable zoonotic gastrointestinal infection after campylobacteriosis and cryptosporidiosis [4]. In 2021, the notification rate of *Salmonella* infections in the EU/EEA was 15.7 per 100,000 population, and 773 food-borne salmonellosis outbreaks were detected [3]. In Finland, *Salmonella* outbreaks are usually small in size, an average of 32 cases per outbreak were reported in 26 outbreaks from 1 January 2010 to 31 December 2020 [5]. In 2021, however, the largest *Salmonella* Typhimurium outbreak in the EU, with more than 700 cases, occurred in Finland [5]. *Salmonella* can be transmitted by food or water contaminated with faeces from animals or humans carrying the bacterium, or more rarely from person to person [1].

In 2021, the clinical microbiology laboratories in Finland reported 474 salmonellosis cases to the National Infectious Disease Registry (NIDR), equating to an annual incidence of nine per 100,000 population [6]. Of these, 407 clinical isolates were sent to the Finnish Institute for Health and Welfare (THL) for further characterisation. Among these isolates, 282 (69%) were from cases who did not report any travel abroad within the incubation period of *Salmonella* infection, indicating that the infections were acquired domestically. Origin information was missing for 120 (29%) isolates. Domestic *Salmonella* infections were caused by 46 different serotypes. The most common serotypes were Typhimurium (n = 190; 47%), Enteritidis (n = 64; 16%), the monophasic variant of *S*. Typhimurium (n = 23; 6%) and Poona (n = 15; 4%). Together, these accounted for 72% of infections. Whole genome sequencing (WGS) was performed for 331 of the 474 (70%) *Salmonella* isolates [6].

## Outbreak detection

In late May 2022, the United Kingdom (UK) reported a cluster of 31 *S*. Mbandaka sequence type 413 (ST413) cases with specimen collection dates between 24 September 2021 and 23 April 2022 [7]. After this, the Expert Microbiology Unit of THL reported an increased number of domestic *S*. Mbandaka cases from different parts of Finland. Ten cases were identified with sampling dates between 19 April and 24 May 2022. A national outbreak investigation was initiated to determine the magnitude and source of the outbreak to prevent further cases and similar outbreaks in the future.

We present our outbreak investigation as a continuum to the joint rapid outbreak assessment by the European Centre for Disease Prevention and Control (ECDC) and European Food Safety Authority (EFSA) [7]. We detail the case–case study using a list of food images, national food consumption data, purchase information, WGS analysis, traceback investigations and collaboration with Estonian authorities, through which we identified the source of the international *S*. Mbandaka outbreak.

## Methods

### Epidemiological investigation

#### Outbreak case definition

A case was defined as a person with laboratory-confirmed *S*. Mbandaka infection notified to the NIDR between 19 April 2022 and 16 January 2023. Clinical laboratories notify laboratory-confirmed salmonellosis cases to the NIDR, a nation-wide electronic reporting system for communicable diseases. Notification data includes age, sex, region of residence, travel status, and sampling date. The treating physician determined the travel status of a case. Domestic cases had not travelled abroad within the incubation period for *Salmonella* infection. 

#### National food consumption data

The THL collects regularly nationally representative data on food consumption and eating habits of Finnish adults. In 2017, this National FinDiet survey was carried out in cooperation with the FinHealth 2017 survey at 50 locations across Finland [8,9]. The methodology consisted of a health examination and two non-consecutive 24 h dietary interviews for adults aged 18–74 years (n = 1,655) [8]. We used data on the percentage of meals consumed outside the home (restaurant or take-away) and on the proportion of users of the following foods: poultry, hamburgers, meat cuts, sausages, as well as vegetables and fruits consumed without heating.

#### Trawling interviews, purchase information data, and traceback investigation

Cases were interviewed by local, regional, or national healthcare professionals. Information was gathered on disease characteristics and food and on environmental exposures within 2 weeks before onset of symptoms using a standardised online trawling questionnaire for food- and water-borne diseases. We compared the food exposure data from trawling interviews of cases reported between 19 April and 12 September 2022 (n = 50) with data from the national food consumption survey. We calculated odds ratios (OR), 95% confidence intervals (CI), and p values for exposures using Fisher's exact test. A p value of less than 5% (i.e. p < 0.05) was considered statistically significant.

We obtained purchase information for 21 cases via their supermarket loyalty cards, aiming to identify any shared products purchased within 2 weeks before the onset of symptoms. We analysed the gathered data using Excel.

We conducted a traceback investigation of the ingredients of the food products identified through the trawling interviews and purchase information analyses.

#### Case–case study

A case–case study was conducted to establish the vehicle of the outbreak. We included in the study *S*. Mbandaka cases reported from September 2022 to January 2023. This period was selected to minimise potential recall bias associated with extended timeframes, as cases and control-cases were contacted in January. A case was defined as described above, but with a notification to the NIDR between 1 September 2022 and 16 January 2023. A control-case (hereafter referred to as control) was defined as a person who had laboratory-confirmed *Salmonella* other than serotype Mbandaka, during the same time period and in the same or neighbouring hospital district as a case. No sample size calculation was performed since all salmonellosis cases reported to NIDR during that time and from those hospital districts were included in the case–case study.

On 23 and 26 January 2023, the cases and controls were contacted by letter and invited to participate in an online questionnaire. The survey included questions about demographics (age and sex), symptoms (date of onset, date of recovery, type of symptoms, hospitalisation), and consumption of 15 ready-to-eat (RTE) products containing chicken, as well as the place of purchase. Of the products, four were RTE products manufactured by Company A in Estonia and contained a chicken meat product originating from the same source as the batch from which *S*. Mbandaka was isolated in December 2022 (see *Microbiological investigations*). Another product included in the questionnaire was also manufactured by Company A but did not contain the chicken meat product from the implicated source. Ten additional products, which were not suspected to be the cause of the outbreak, were included as product controls.

To assist recall, the questionnaire included pictures of the RTE products containing chicken. Participants were asked to mark the products they had consumed in the 7 and 30 days before symptom onset. Participants who had not marked a given product were considered unexposed. The participants who responded probably having eaten or probably not having eaten a specific product were grouped as having eaten or not having eaten it, respectively. In addition, those who selected having eaten a product in the 7 days before symptom onset were accounted for as also having been exposed in the previous 30 days.

A univariate analysis of exposure to these products was conducted using Fisher’s exact test. We calculated product-specific ORs and their 95% CIs for single and combined exposures, the latter being the five suspected products combined. A p value of less than 5% was considered statistically significant. For the purposes of calculating a rough OR estimate, in the case of products for which there were no exposed controls, one person was added to each group (i.e. exposed case, unexposed case, exposed control, unexposed control). The analysis was performed using RStudio software (R version 4.2.1).

#### Outbreak investigation in Estonia

Our investigation centred on the Estonian Company A which produced and supplied RTE products to Finland and was implicated in the survey responses. In September 2022, THL alerted the Estonian Agriculture and Food Board of an ongoing *S.* Mbandaka outbreak. In response, an investigation was initiated in Estonia retrospectively to identify possible cases during 2022. The patient samples were sequenced at the Estonian Veterinary and Food Laboratory and the sequences were shared with Finland to be compared with the sequences of the Finnish cases. 

### Microbiological investigation

In Finland, samples were provided by individuals tested for *Salmonella*, most often due to symptoms of gastrointestinal illness, but also in some cases as part of routine screening (e.g. for work in food premises). Testing was conducted at clinical laboratories using culture and/or PCR. *Salmonella* PCR-positive samples were cultured, and isolates from the culture-positive samples were sent to the THL reference laboratory. Clinical laboratories routinely send the following *Salmonella* isolates to THL for further analysis, including serotyping, antimicrobial susceptibility testing, and WGS: (i) isolates from blood samples, (ii) isolates from *S*. Typhi and *S*. Paratyphi, and (iii) isolates from domestically acquired infections. All isolates in this study were serotyped using the slide agglutination method [10]. We performed WGS using the Nextera XT DNA Library Preparation Kit and MiSeq sequencer (Illumina Inc). Cluster analysis from sequence data was performed by core-genome multilocus sequence typing (cgMLST) using Ridom SeqSphere+ software, version 9.0.10 (2023–09) [11]. The allele thresholds and cluster criteria were established retrospectively after the initial cases were identified and sequencing data became available. These criteria were determined based on the cgMLST scheme, the number of isolates in the comparison, and available epidemic data (e.g. time and place).

The strain isolated from the RTE-product containing chicken was first sent for typing by slide agglutination to the laboratory of the Finnish Food Authority (Kuopio) and from there to THL for WGS.

## Results

### Epidemiological investigation

**Figure 1:**
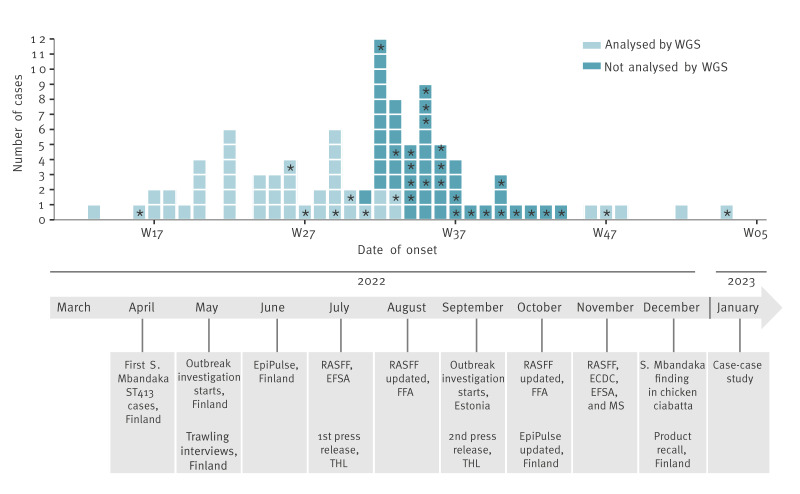
Laboratory-confirmed *Salmonella* Mbandaka cases by date of symptom onset, Finland, March 2022–January 2023 (n = 97)

ECDC: European Centre for Disease Prevention and Control; EFSA: European Food Safety Authority; FFA: Finnish Food Authority; MS: European Union member states; RASFF: Rapid Alert System for Food and Feed; THL: Finnish Institute for Health and Welfare; WGS: whole genome sequencing.

We identified 97 cases in Finland between 19 April 2022 and 16 January 2023 (Figure 1). The cases’ median age was 27 years (range 0 to 74 years); 61% (n = 59) were female. Cases occurred in 18 of 21 hospital districts in Finland.

* The asterisk marks cases with an unknown date of symptom onset or who were asymptomatic, for whom date of sample collection was used. A timeline below the graph illustrates the key events during the outbreak.

Of the 97 cases, 78 (80%) were interviewed by the trawling questionnaire. Of them, 76 (97%) had not been travelling abroad in the week before symptom onset. The most common reported symptoms were diarrhoea (60/78; 77%), abdominal pain (55/78; 71%), nausea (43/78; 55%), fever (34/78; 44%), bloody diarrhoea (21/78; 27%), and vomiting (12/78; 15%). Urinary tract infection was reported by 15 (19%) cases. Seven (9%) cases were asymptomatic. Twelve (15%) were hospitalised, with stays ranging from 24h to 13 days (mean of 3 days). Six of these cases developed septic infections. No deaths were reported.

#### National food consumption data, purchase information, and traceback investigation

For hypothesis generation, we compared exposure data from the trawling interviews and the National FinDiet 2017 survey. Results showed that, compared with the general population, cases were more likely to have eaten at a restaurant or takeaway, or to have consumed poultry products, or hamburgers (Table 1). Moreover, based on the interview data, out of the total 78 interviewed cases, 72 (92%) had consumed (n = 66) or possibly consumed (n = 6) chicken in various forms. We traced back chicken products linked to 12 restaurant servings and one RTE product sold at retail and mentioned by the cases in the interviews. The products originated from several manufacturers located in different non-EU and EU countries, including Finland. Of the interviewed cases, 21% (16/78) had consumed RTE products containing chicken produced by Company A during the recall period.

**Table 1:** Food exposures reported by *Salmonella* cases in trawling interviews, April–September 2022 (n = 50), compared with food consumption among the adult population in the National FinDiet 2017 survey (n = 1,655), Finland

Exposure	Trawling interviews			FinDiet 2017			OR (95% CI)	p value
	Total cases	Exposed		Total controls	Exposed			
		n	%		n	%		
Dining in a restaurant/take-away	50	43	86	23,636^a^	1,200	5	115 (52–256)	< 0.001
Poultry	50	48	96	1,655	766	46	28 (7–115)	< 0.001
Hamburgers	50	24	48	1,655	183	11	7 (4–13)	< 0.001
Cold cuts	50	45	90	1,655	1,192	72	3.5 (1.4–9)	0.005
Sausages	50	21	42	1,655	504	30	1.7 (0.9–3)	0.081
Unheated vegetables	50	42	84	1,655	1,508	91	0.5 (0.2–1.1)	< 0.001
Unheated fruits	50	29	58	1,655	1,348	81	0.3 (0.2–0.6)	< 0.001

CI: confidence interval; OR: odds ratio.

^a^ Total number of meals in FinDiet 2017 data (n = 23,636).

Cases (n = 50) with date of symptom onset from 19 April 2022 to 12 September 2022, aged 18 years or older were included in the analysis. Fisher’s exact p values < 0.05 were considered as significant.

**Figure 2:**
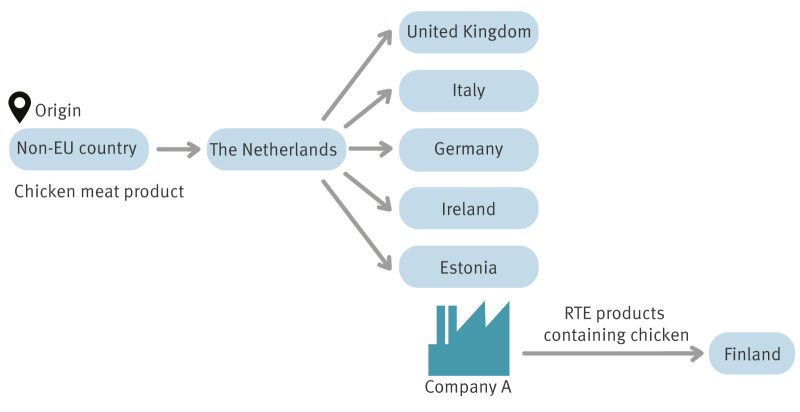
Traceback diagram of ready-to-eat products containing chicken, the suspected source of the *Salmonella* Mbandaka outbreak, Finland, 2022–2023

EU: European Union; RTE: ready-to-eat.

Further investigations identified that pre-cooked chicken breast fillet used in a ciabatta product was positive for *S.* Mbandaka and originated from a non-EU country. The distributor of the chicken breast fillet was a company based in the Netherlands. In January 2023, the Netherlands alerted EU countries via the Rapid Alert System for Food and Feed (RASFF) that the same chicken breast fillet had been distributed to EU countries including Estonia, Italy, Germany and Ireland, as well as the UK (Figure 2).

Arrows indicate the direction of distribution from the product’s origin to the consumers in Finland.

We obtained purchase data from 17 of the 21 cases who had supermarket loyalty cards. Eleven of the 17 had bought RTE products containing chicken within 2 weeks before symptom onset. Of these 11 cases, three had purchased chicken wraps and two chicken ciabattas from Brand A, while four had purchased chicken ciabattas from Brand B. Brands A and B were produced in the same production plant by Company A.

#### Case–case study

Questionnaires were sent to 23 cases and 46 controls. Responses were received from nine cases and 15 controls. No statistically significant differences were observed between cases and controls in terms of age, sex, or hospitalisation. A wrap product from Brand A and a ciabatta from Brands A and B presented the highest odd ratios for *S.* Mbandaka infection (Table 2). In line with this, for persons eating at least one of the products from Company A, the odds of illness were greater than for persons unexposed to these products (Table 2).

**Table 2:** Food products consumed before onset of symptoms, *Salmonella* Mbandaka case–case study, Finland, September 2022–January 2023 (n = 24)

Product	Exposed among (n = 9) cases		Exposed among (n = 15) controls		OR (95% CI)	p value
	n	%	n	%		
Consumed 7 days before symptom onset						
Wrap (Brand A)	4	44	0	0	13 (1.3–139)^a^	0.012
Ciabatta (Brand A)	2	22	0	0	Inf (0.3–Inf)	0.130
Ciabatta (Brand B)	2	22	0	0	Inf (0.3–Inf)	0.130
Ciabatta (other)	0	0.0	2	13	0.0 (0.0–9)	0.511
Sandwich (Brand C)	2	22	1	7	4.0 (0.2–252)	0.533
Sandwich (other)	2	22	2	13	1.9 (0.1–30)	0.615
Wrap (Brand C)	0	0	1	7	0.0 (0.0–65)	1.000
Wrap (other)	1	11	1	7	1.8 (0.0–147)	1.000
At least one product of brands A or B produced by Company A	6	67	1	7	28 (1.9–1,361)	0.004
Consumed 30 days before symptom onset						
Wrap (Brand A)	4	44	0	0	13.3 (1.3–139)^a^	0.012
Ciabatta (Brand B)	4	44	0	0	13.3 (1.3–139)^a^	0.012
Ciabatta (Brand A)	3	33	0	0	9.1 (0.9–97)^a^	0.042
Ciabatta (other)	0	0	2	13	0.0 (0.0–9)	0.511
Sandwich (Brand C)	2	22	1	7	4.0 (0.2–252)	0.533
Sandwich (other)	2	22	2	13	1.9 (0.1–30)	0.615
Wrap (Brand C)	0	0	1	7	0.0 (0.0–65)	1.000
Wrap (other)	1	11	1	7	1.8 (0.0–147)	1.000
At least one product of Brands A or B produced by Company A	7	78	1	7	49 (3–2,308)	0.001

CI: confidence interval; Inf: infinite; OR: odds ratio.

p values < 0.05 were considered as significant.

^a^ 1 added to all groups: originally, OR ∞.

#### Outbreak investigation in Estonia

Four laboratory-confirmed cases of *S*. Mbandaka were detected in Estonia with dates of sampling between 26 April and 21 September 2022. Two of them had a travel history to Finland. 

### Microbiological investigation

**Figure 3:**
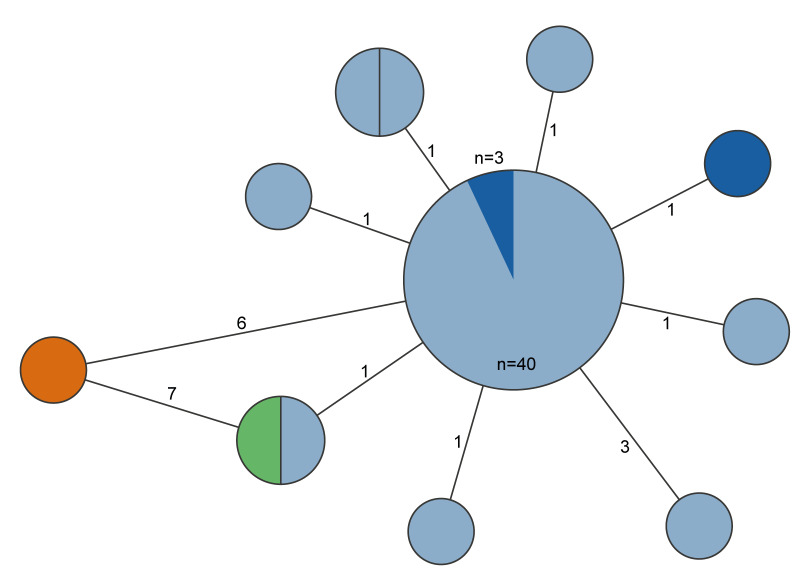
Minimum spanning tree of *Salmonella* Mbandaka isolates based on core-genome MLST analysis, Finland, reporting date April 2022–January 2023 (n = 54)

Between 19 April 2022 and 16 January 2023, isolates from 97 cases were identified as *S.* Mbandaka with antigenic structure 6,7:z10:e,n,z15. All sequenced Finnish human isolates (n = 48) were typed as MLST ST413. For 49 isolates with sampling dates between August and October 2022, WGS was not performed due to limited laboratory resources during the summer and early autumn (see Figure 1). In the cgMLST analysis of 1,261 target alleles (after excluding 162 missing alleles), all sequenced Finnish human isolates were found to be closely related, with a maximum of three allelic differences. In addition, Finnish isolates were linked to the Estonian cases by a maximum of one allelic difference (Figure 3).

MLST: multi-locus sequence typing.

The tree was generated using Ridom SeqSphere+ based on 1,261 alleles, with no missing values. All isolates belonged to sequence type ST413. Depicted are human isolates from Finland (n = 48) and Estonia (n = 4) as light and dark blue, respectively. Food isolates (n = 2) from a suspected product produced by Company A (in orange) and a chicken product used as its ingredient (in green) are also shown. Numbers indicate allelic differences between isolates.

In December 2022, Finnish Customs detected *S.* Mbandaka in a suspected product manufactured by Estonian Company A (i.e. a chicken ciabatta product, represented in orange in Figure 3). This isolate was classified as MLST ST413, consistent with the sequence type of the Finnish human isolates. Upon receiving this information, Company A conducted additional environmental sampling and tested the chicken product used as an ingredient in the chicken ciabatta. *Salmonella* Mbandaka was isolated from the pre-cooked chicken breast fillet used in this product (depicted in green in Figure 3). Subsequent WGS analyses revealed that this isolate clustered with *S*. Mbandaka strains found in Estonian and Finnish cases, exhibiting one allelic difference from the main cluster (Figure 3).

### Outbreak control measures

On 16 June 2022, we published an EpiPulse enquiry via the Food and Waterborne Diseases Network (FWD-Net) of the ECDC to chart the incidence of *S*. *enterica* serotype Mbandaka ST413 in other European countries. This revealed cases of *S.* Mbandaka ST413 in Czechia, Estonia, France, Germany, Ireland, the Netherlands, the UK, and Israel [7]. On 29 July 2022, the European Food Safety Authority (EFSA) opened a RASFF notification (News 2022.4440) to inform the food safety authorities of an ongoing multi-country outbreak. We published two press releases, on 20 July and 2 September, to inform healthcare professionals and the public about the outbreak investigation in Finland (Figure 1). On 26 August, we followed up the RASFF with a traceability analysis of RTE products containing chicken consumed by the cases, and on 10 October, with Finnish cases’ consumption of Company A’s products. On 23 December, a Finnish company that imported the contaminated product recalled the chicken ciabatta following preliminary detection of *Salmonella* spp. (Figure 1). Subsequently, on 30 December, the Finnish Food Authority notified RASFF (2022.7654) of the presence of *S.* Mbandaka in the product.

Following the investigations in Estonia, the Estonian Agriculture and Food Board made updates to RASFF 2022.7654, providing additional information regarding the detection of *S*. Mbandaka in food items in early 2023, and recalled the pre-cooked chicken breast fillet of non-EU origin.

## Discussion

A multi-country outbreak of *S*. Mbandaka, spanning from September 2021 to January 2023, resulted in over 200 cases, approximately half of them occurring in Finland. We integrated information from trawling interviews, purchase records and national food consumption data, indicating a potential link between the outbreak and the consumption of RTE products containing chicken. A case–case study conducted in January 2023 allowed us to establish the consumption of specific products from Company A as the most likely source of *S*. Mbandaka infections. In support of these findings, *S*. Mbandaka was isolated from both cases and food samples. All Finnish case isolates were closely related to each other, to case strains shared by the Estonian and UK authorities [7], and to the strain isolated from the chicken meat used as an ingredient by Company A.

Our results suggest that the pre-cooked, frozen chicken meat product used by Company A was the source of the multi-country outbreak of *S*. Mbandaka. The traceback investigation showed that the meat product originated from a non-EU country and transited through the Netherlands to Estonia, where it was used by Company A to manufacture RTE products. Company A distributed these RTE products solely to Finland. In addition to Estonia, the chicken meat product had also been distributed to other countries where human cases of *S.* Mbandaka infections had been reported, including Germany, Ireland, Italy and the UK [7]. After Company A discontinued the use of the contaminated chicken meat product and ceased operations with the implicated meat supplier, no new cases attributed to the outbreak were detected in Finland for 9 months.

In October 2023, however, a case of *S.* Mbandaka exhibiting the same outbreak strain was reported in Finland. The case had purchased a chicken baguette produced by Company A in early October 2023. Subsequent internal sampling efforts by Company A (i.e. raw materials, RTE products, and environmental samples for *Salmonella* spp. detection) yielded no evidence of *Salmonella* contamination. Few studies have addressed the biology of *Salmonella* persistence in humans and the virulence mechanisms required for long-term infection. While persistent infections are more commonly associated with cases of typhoid fever caused by *Salmonella* Typhi [12], long-term carriage of non-typhoidal *Salmonella* (NTS) is exceedingly rare. However, temporary carriage of NTS has been reported, with individuals shedding the bacteria for up to a year (reviewed in [12-14]). Among NTS, *S*. Mbandaka is notably associated with persistent salmonellosis [14]. Nevertheless, attributing long-term carriage to an isolated case remains speculative. While the original outbreak appears conclusively resolved, the emergence of this case underscores the importance of continuous surveillance.

Outbreaks caused by *Salmonella* and associated with contaminated food products have been reported in the past in the EU/EEA and the UK [3,15]. In these countries, *S*. Mbandaka has been frequently isolated from cattle, chickens, and animal feed [16,17] but rarely linked to human outbreaks [18,19]. *Salmonella* Enteritidis retains its status as the predominant serovar reported in human cases, exhibiting associations with various sources, particularly broilers and laying hens [3]. In Finland, *Salmonella* outbreaks have mostly been caused by imported vegetables or broiler meat [20,21]. Elsewhere, eggs and egg products have been identified as the principal source of salmonellosis outbreaks [22]. Prophylactic measures to prevent salmonellosis outbreaks address all stages of the food supply, from production to distribution and consumption. Poultry vaccination and improved hygiene through measures in production and distribution have achieved a reduction in human illnesses [23]. Other prevention-oriented behaviours include avoiding or safely preparing food and beverages that could be contaminated, as well as hand washing with soap [1].

As for Finland, the European Commission approved a *Salmonella* control programme in the context of Finland's accession negotiations at the end of 1994, and it was introduced as a national programme from 1995 [24]. Under this programme, *Salmonella* control in poultry and egg production is carried out throughout the supply chain. The control programme provides Finland with an added safeguard against *Salmonella*, requiring that imported raw meat, minced meat, and flocks of origin for eggs test negative for *Salmonella* in the country of origin. However, pre-cooked frozen chicken products are not covered under this testing requirement. In case of suspected *Salmonella* contamination or possible outbreaks, the food authorities should be informed, the food business operator should suspend the use of the suspected products and those should be tested [21]. In addition, if necessary, the company must proceed with the withdrawal of the product from the market.

Our study had some limitations. Firstly, not all isolates from *S.* Mbandaka cases with date of onset between August and October 2022 were whole-genome sequenced and could therefore have been misclassified as outbreak-related. However, isolates from four additional cases occurring in December 2022 and one in January 2023 were sequenced to make sure that they were part of the cluster (Figure 1). All belonged to the same genotype and clustered with outbreak isolates, indicating that the non-sequenced cases from the earlier period most probably belonged to the same cluster. In Finland, *S*. Mbandaka is a rare serotype, with annual reported cases ranging from none to seven between 2016 and 2021, supporting the assumption that the cases identified in 2022 were part of the outbreak. Secondly, difficulties with recall must be considered in the case–case study, as the questionnaire was sent out 4–5 months after the possible exposure of most participants. To mitigate this recall difficulty, pictures of the suspected products were added to the case–case study questionnaire and only the most recent cases were recruited. Finally, the number of cases and controls (i.e. control-cases) recruited and included in the case–case study was relatively small, as only about one third of cases and controls responded. Despite these limitations, the results of the epidemiological and microbiological investigations, together with the traceback of products containing chicken, pointed towards coherent findings.

We could not confirm the exact point of contamination in the meat production chain. Although the chicken meat used was pre-cooked and frozen, the chicken could have been cooked improperly or contaminated afterwards. In fact, previous outbreaks have been associated with frozen meals, demonstrating that *Salmonella* may survive freezing temperatures [25]. In Finland, outbreaks of *Salmonella* have been previously associated with frozen tomato cubes and frozen, pre-cooked chicken cubes [21,26]. Furthermore, *Salmonella* was not detected at the first inspection in Company A, but later. This could be due to the fact that *Salmonella* can be unevenly distributed in the batches, and low levels or sporadic contamination may not be detected in the sampling [21].

In many cases, obtaining conclusive evidence of the relationship between food sources and human infections poses a challenge. Systematic and continuous surveillance of animals and their food products, both on-farm and at retail, could be of great value in detecting the presence of *Salmonella*. This could also contribute to improved interventions aimed at decreasing *Salmonella* isolation rates in foods of animal origin throughout the food chain. Ensuring that pre-cooked meat products used in RTE products are properly cooked during processing is a critical control point. Visual inspection of each batch by food business operators, especially when the RTE meat product is used without further reheating, may help identify improperly cooked batches. 

This outbreak investigation was the result of a well-functioning collaborative effort involving two EU countries, Finland and Estonia, with support from EpiPulse of the ECDC [27]. This underscores the pivotal role of cross-border cooperation in addressing and mitigating such events. Furthermore, we recommend the use of case–case studies, particularly when the selection of controls can be cumbersome. This approach selects controls from a subpopulation equally likely to seek medical attention and undergo pathogen sampling, which helps reduce selection bias. In addition, such studies offer the benefit of minimising recall bias, given that both cases and control-cases have experienced symptoms, enhancing the overall robustness of the investigation [21,28,29]. In our case–case study, we also used food images, which proved useful in identifying consumed products months after illness onset.

## Conclusions

This outbreak investigation highlights the public health value of timely, cross-sectoral collaboration across borders. Close cooperation between Finland and Estonia, supported by EU platforms, was crucial in identifying and controlling the source of infection. Our findings also reinforce the utility of case–case studies as an effective epidemiological tool in outbreak investigations, especially when traditional control selection poses challenges. In addition, the use of food images proved helpful in supporting recall during interviews conducted months after symptom onset. These approaches may strengthen future outbreak responses by improving the accuracy of exposure assessment and facilitating timely identification of foodborne sources.
